# Prospect of cell penetrating peptides in stem cell tracking

**DOI:** 10.1186/s13287-021-02522-3

**Published:** 2021-08-14

**Authors:** Xiaoshuang Zhang, Tong Lei, Hongwu Du

**Affiliations:** 1 Daxing Research Institute, University of Science and Technology Beijing, Beijing, 100083 China; 2 School of Chemistry and Biological Engineering, University of Science and Technology Beijing, Beijing, 100083 China

**Keywords:** Stem cell tracking, Cell penetrating peptides, MRI, Fluorescence imaging, Ultrasound imaging

## Abstract

Stem cell therapy has shown great efficacy in many diseases. However, the treatment mechanism is still unclear, which is a big obstacle for promoting clinical research. Therefore, it is particularly important to track transplanted stem cells in vivo, find out the distribution and condition of the stem cells, and furthermore reveal the treatment mechanism. Many tracking methods have been developed, including magnetic resonance imaging (MRI), fluorescence imaging, and ultrasound imaging (UI). Among them, MRI and UI techniques have been used in clinical. In stem cell tracking, a major drawback of these technologies is that the imaging signal is not strong enough, mainly due to the low cell penetration efficiency of imaging particles. Cell penetrating peptides (CPPs) have been widely used for cargo delivery due to its high efficacy, good safety properties, and wide delivery of various cargoes. However, there are few reports on the application of CPPs in current stem cell tracking methods. In this review, we systematically introduced the mechanism of CPPs into cell membranes and their advantages in stem cell tracking, discussed the clinical applications and limitations of CPPs, and finally we summarized several commonly used CPPs and their specific applications in stem cell tracking. Although it is not an innovation of tracer materials, CPPs as a powerful tool have broad prospects in stem cell tracking.

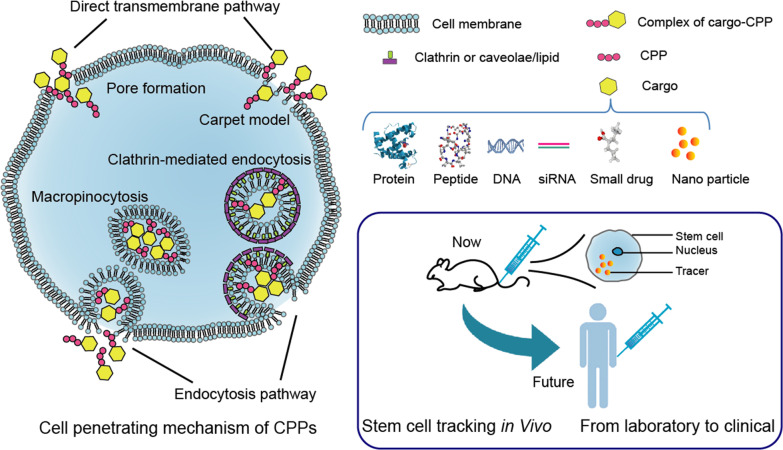

## Introduction

Stem cell therapy is a potential method for multiple diseases in future clinical treatment since the current experimental data are mostly positive [1–7]. However, there are few clinical reports related to stem cells. Although the excellent potential stem cells have shown in human disease, they also can be dangerous if used incorrectly. Since the accumulation of stem cells in the wrong place may cause safety issues, the possibility of tumorigenesis is also a hidden danger [8]. Therefore, it is necessary to track the transplanted stem cells and further determine the safety and effectiveness of stem cell therapy.

Stem cell tracking is an effective method to monitor transplanted stem cells in vivo, by which we aim to clarify the distribution and situation (cell viability and differentiation) of stem cells. To achieve the above goals, tracking methods have some rules to follow: Firstly, the trackers have to be low-toxic to cells, not affect cell properties. Besides, ideal trackers need to have high labeling efficiency and emit strong detection signal to form high resolution image. The last point is the stableness and specificity of trackers. In recent years, many in vivo cell tracking methods have been developed. There are two main strategies for stem cell tracking. One is to transfect the genetically modified reporter genes into the stem cells [9–13], and the other is labeling the cells with imaging particles [14–16]. However, reporter gene labeling needs complicated gene modification and transfection steps which may cause concern of the safety in clinical application [17]. The tracking method labeling with imaging particles is relatively feasible which includes MRI, X-Ray computed micro tomography (micro-CT), positron emission tomography (PET), UI, and FI. Existing detection technologies mainly rely on optics, magnetism, and radiology. However, these tracking methods cannot meet all the requirements of stem cell tracking. Thus, modifying imaging particles to improve tracking effect has become a trend. One strategy is to decorate signal emitting particles with a higher cell labeling efficiency molecular, like CPPs.

Since the discovery of trans-activator of transcription (TAT) is derived from human immunodeficiency virus type 1 (HIV-1) in 1980s, many peptides with the ability to penetrate cell membrane have been added to the CPPs family [18–20]. CPPs have played an important role in delivery systems with a variety of cargos, such as nucleic acids, polymers, liposomes, nanoparticles, and low molecular weight chemical drugs [21]. These cargoes can be connected to the CPPs in a covalent or non-covalent manner. The latter is connected through electrostatic interactions, in which the activity of the cargoes can be better maintained. In addition to the wide delivery of various cargoes mentioned above, CPPs also have the advantages of natural low cytotoxicity and high penetration efficiency in various cell types [22–24]. These advantages make CPP widely used in basic and clinical research. However, there are few reports on the applications of CPPs in stem cell tracking. Therefore, this review aims to introduce CPPs for stem cell tracking.

## Cell penetrating peptides (CPPs)

CPPs are positively charged short peptides with 5–30 amino acids [22]. The possibility of penetrating into biological membrane makes CPPs become novel carriers for intracellular cargo delivery [23, 25–29]. Compared with chemical molecules, CPPs have lower biological toxicity and higher transduction efficiency [30, 31]. Overall, delivery efficiency of the CPPs may be depending on some parameters such as the size of cargo-CPP complex, nature of CPPs, and so on. In this review, we introduced different kinds of CPPs and how they can be used in stem cell tracking, including existing applications and future possibility.

### Classification of CPPs

Based on their origin, CPPs are classified as protein-derived, chimeric, and synthetic substance. CPPs were originally obtained from natural products and most of the CPPs were derived from natural proteins, including DNA/RNA-binding proteins, antimicrobial peptides, and heparin-binding proteins [32–34]. Chimeric CPPs referred to CPPs derived from a combination of natural proteins or peptides, such as Transportan and Pep1 [20, 35]. A series of synthetic CPPs were subsequently developed to mimic natural protein-derived CPPs, like model amphipathic peptide (MAP) [36].

The other classification is based on the physical–chemical properties of CPPs by which CPPs were divided into cationic, amphipathic, and hydrophobic peptides. The first two types are more common as carriers in particle modification. According to the properties of the particles, appropriate types of CPPs were selected.

### Cell penetrating mechanism

The precise cell penetrating mechanism of CPPs was not clear, but it seems that this uptake pathway is energy independent [37]. The penetrating mechanism of CPPs seems to depend not only on the type of CPPs, but also on its concentration, the cargo being transported, and the targeted cells [38]. For example, the internalization of the TAT/fusion protein complex is mainly limited to endocytosis, but the TAT/small peptide complex is internalized by endocytosis and rapid direct membrane translocation [39]. Several current cell penetration theories are based on specific CPPs.

Direct transmembrane transport is similar to the simple diffusion of small molecules. One theory explained transfer mechanism of cationic CPPs, that is, the cationic peptide and the negatively charged membrane component can form a transient ion-pair complex, then the reduced but still positively charged peptide makes it diffuse adaptively through the cell membrane, where the driving force for diffusion is membrane potential [40]. This membrane penetration method is described as the “carpet-like” model. The formation of transient toroidal pores is considered as another possible mechanism for direct translocation. At high concentrations of TAT peptide, transient pores may be formed in the membrane since TAT peptide may interact with lipid bilayer to form molecular dynamic effect [41]. The model shows that the TAT peptide has phospholipid head groups on both the outer and inner lipid leaflets, which causes a thinning of the lipid bilayer and eventually penetrate into the membrane.

Although direct transfer of cell membranes happens in some cases, it is generally believed that most CPPs and their cargo enter the cell by endocytosis. This manner includes clathrin-mediated endocytosis, caveolae/lipid raft-mediated endocytosis, and micropinocytosis [42–45]. Since these are relatively mature mechanisms, they will not be discussed here.

## Advantages of CPPs in stem cell tracking

Due to its biological characteristics, CPPs have been widely used in a variety of fields, including drug delivery [23, 28, 29], anti-bacterial infection [46, 47], and gene delivery [25–27]. And these characteristics of CPPs also make it advantageous in tracking transplanted stem cells.

### Low cytotoxicity

Low toxicity at working concentration is the first consideration for the application of tracking particles to stem cells. The cytotoxicity mentioned here includes proliferation toxicity and multi-lineage differentiation toxicity, that is, the effect on the stemness of stem cells. CPPs can enter cells without causing cytotoxicity at the working does [22]; therefore, they have been used as a tool for the delivery of various cargoes [48–50]. Especially in stem cell tracking experiments, CPPs-modified imaging particles did not affect the differentiation potential of stem cells in vitro [27, 51, 52].

### Transfer efficiency /does-dependent efficiency

The biggest advantage of CPPs as delivery molecules is the transfer efficiency. Moreover, the cargo it delivered has little effect on the penetration ability of CPPs, although the manner of transmission may change [23]. Compared with original imaging particles, the internalization rate of CPPs-connected imaging particles is increased by 2–200 times [51–53]. In this way, the labeling rate of cells can reach more than 80%.

### Effectiveness on variety of cell types

Although in clinical practice we are increasingly advocating personalized therapy to achieve more precise treatment, in terms of cell labeling, it is a common wish to use a universal method to label various types of cells. It has been proved that CPPs have transmembrane effect on multiple cells [54–56]. On the same platform, Gillmeister et al. [24] reported that rhodamine-labeled TAT-GFP was internalized in multiple cell lines including HEK293, N18-RE-105, hippocampal slices, and human neural progenitor cells and showed predominantly endosomal localization of both fluorescent markers.

### Easy to conjugate

There are two ways to conjugate CPPs to the cargo: covalent and non-covalent binding [22] Although both methods are feasible, in practical applications, the second method is more popular [57]. In non-covalent approach, CPPs bind to cargo through electrostatic interaction [29]. Due to its high flexibility, this method is suitable for a wide range of cargo delivery applications. More importantly, this method avoids the trouble of unique conjugation design for different cargoes.

### Wide variety of cargoes

It has been extensively shown that CPPs are capable of transporting into cells a wide variety of biologically active cargoes including magnetic nanoparticles, proteins, peptides, DNAs, mRNAs, siRNAs, and small drugs [21, 58, 59]. This feature makes CPPs a universal delivery vector and plays a role in a variety of cell tracking technologies, effectively improving the labeling efficiency of various tracer particles.

## CPPs in current clinical study

### Safety and effectiveness in clinical application

Due to its excellent performance in animals, CPPs have also been widely used clinically in recent years. We have compiled some reports on the clinical application of CPPs (Table 1). In these reports, there were no adverse reactions caused by CPPs. Regarding the effectiveness of CPP, since there is no design of a drug without CPP coupling as a control in clinical reports, the experimental results can only prove the effectiveness of the CPP-cargo complex.

**Table 1:** CPP-conjugated therapeutics in clinical trial

Disease	CPP-cargo	Number of enrolled patients	Status	ClinicalTrials.gov ID
Solid tumors	P28	*N* = 15	Phase I completed 2014	NCT00914914
CNS malignancies	P28	*N* = 18	Phase I completed 2017	NCT01975116
Postherpetic neuralgia	TAT-ɛPKC inhibitor	*N* = 23	Phase II completed 2011	NCT01106716
Acute inner ear hearing loss	TAT-AM 111	N = 256	Phase III completed 2017	NCT02561091
Tumor imaging	ACPPs-Cy5 and Cy7	*N* = 27	Phase I completed 2017	NCT02391194
Cervical dystonia	TAT-Daxibotulinumtoxin A	*N* = 37	Phase II completed 2019	NCT02706795
Postoperative ocular inflammation	TAT-XG102	*N* = 339	Phase III completed 2017	NCT02235272

TAT, as a well-researched CPP, is relatively common in clinical applications. In a clinical trial, the synthetic peptide KAI-1678 (containing 21 amino acids) was used to treat postherpetic neuralgia. This polypeptide contains the active site of the inhibitor of ɛPKC enzyme and the CPP (TAT). In order to enhance the stability of peptides in vivo, acetylated N-terminus and amidated C-terminus were used in the modification of TAT. The results showed that compared with lidocaine, KAI-1678 has good tolerance in vivo, while the analgesic effect is poor [60]. The JNK/c-JUN cascade signaling pathway is a key pathway for chemical and mechanical ear injury [61]. AM-111 is a 31 amino acid dextrorotatory peptide, which contains 19 amino acid effector domains and 10 amino acid active transporter TAT. AM-111 can effectively block the JNK/c-JUN cascade signaling pathway. Clinical experiments have shown that intratympanic injection of AM-111 is used to treat idiopathic sudden sensorineural hearing loss and has a significant therapeutic effect [62]. In the second-phase clinical treatment of cervical dystonia, injection of TAT-coupled daxibotulinumtoxin A can effectively improve the disease and has the potential to provide long-term curative effects. The drug is well tolerated in humans [63]. XG-102 is a TAT-conjugated dextrorotatory peptide that can inhibit c-Jun N-terminal kinase. Clinical studies have shown that a single subconjunctival injection of XG-102 at the end of eye surgery is not inferior to dexamethasone eye drops for postoperative ocular inflammation [64].

In recent years, a new type of synthetic peptide (P28) which contains CPP structure has been used for targeted therapy of tumors. The mutation of the P53 gene in tumors will cause the imbalance of the protein level of P53, which will lead to further tumor expansion. It was reported that cupredoxin azurin can exert anti-tumor activity by targeting wild-type P53. The protein contains both the functional region P28 (amino acids 50–77 of azurin) and the cell penetrating peptide region P18 (amino acids 50–67 of azurin). During the 48-week treatment, the patient tolerated P28 well without significant adverse reactions [65]. In another treatment for adolescent central nervous system tumors, P28 showed good drug tolerance and certain effectiveness [66].

The activatable cell penetrating peptides (ACPPs) is a polypeptide designed on the basis of traditional CPP to exert a penetrating effect in a specific environment. Photo-peptide dye conjugate AVB-620 contains ACPP and fluorescent dyes (CY3 and CY5). Preclinical studies have shown that AVB-620 imaging could display primary tumors in real time, showing high in vivo diagnostic sensitivity and specificity (> 95%) [67, 68]. In clinical trials, AVB-620 was transplanted into the human body through intravenous injection for real-time imaging of breast cancer surgery. The results showed that there were no adverse reactions caused by AVB-620, and AVB-620 improved the detection of malignant tissues during surgery [69].

### Limitations in clinical application

Although there have been many applications in clinical drug delivery, CPP still has some problems and limitations.

#### Cellular uptake mechanism of CPPs is ambiguous

The exact mechanism by which CPP enters the cell is still unclear [22, 70]. Although CPP has been proven to be non-toxic under effective penetrating concentration (generally less than 50 μM) [54, 71–74] at the cellular and animal levels, the effect of CPP on cell function has not been fully evaluated yet. The unclear penetration mechanism will also bring technical barriers to subsequent experiments, such as how to improve penetration efficiency, how to achieve unidirectional transmembrane transport, how to improve stability in the body, and so on.

#### Lack of cell and tissue specificity

Most of the CPPs found so far are positively charged polypeptides, so its ability to penetrate cell membranes is likely to be related to the force between charges [23]. And experiments have shown that a specific CPP can penetrate a variety of cells [54–56]. The above results indicate that CPPs do not have a specific binding response to cells.

This limitation impeded many clinical or preclinical studies that require drug delivery to the target site in order to avoid causing systemic damage. In view of this defect of CPPs in drug delivery, there have been studies to correct it by adding tumor-targeted amino acid sequences [71]. However, there are few specific recognition sites for tumor markers (such as Transferrin, RGD, Lys(3)-bombesin, and NGR) [75, 76], and many kinds of tumor sites need more recognition sequences to be discovered. Another way to increase the target ability of CPPs is to explore ACPPs. This kind of peptides is only activated under certain physiological conditions [77]. For example, ACPPs designed for the weakly acidic physiological environment of the tumor site can target the tumor well, increase the concentration of the drug at the target site, and show a good anti-tumor effect [73]. In addition, some ACPPs are designed to be activated under heat or light conditions [78]. This strategy has broader application and is worth of promoting. However, more subtle targeting requirements for subcellular structures, such as mitochondria or endoplasmic reticulum, are still difficult to achieve by this method. There have been pioneer experiments to study this aspect, and more extensive and in-depth research is needed.

#### The penetration ability to different cells is variable

Studies have shown that different cells have different uptake rates of a specific CPP [54, 55]. The uptake ratio of different CPPs by the same cell is also different [79, 80]. This feature requires that the target cell and the selected CPP need to be determined at the same time in the preclinical experiment, so as to determine the delivery concentration of the CPP.

#### The stability needs to be improved

Traditional CPPs are mostly straight-chain amino acids without steric structure, corresponding to proteolytic instability [78]. In animal experiments, CPPs had a short plasma half-life [21, 71], which became a major drawback in clinical application. However, CPP may be metabolized before being delivered to the target site. In response to this shortcoming, the emerging CPPs in recent years improved the plasma half-life by introducing masking groups or transforming linear CPP into a cyclic (or bicyclic) structure [81, 82].

## Applications of CPPs in stem cell tracking

We summarized the examples of CPPs in stem cell tracing, as shown in Table 2. According to the imaging method, we divide the application of CPPs in stem cell tracking into the following three categories.

**Table 2:** Common CPPs in stem cell tracking application

CPPs	Amino acid sequence	Connected nanoparticles	Cell type	Tracking technology
TAT [66]	RRRQRRKKRG	Gd	hMSCs	MRI
R9 [65]	LAGRRRRRRRRRK	Gd	rBMSC	MRI
TAT [57]	–	Long persistent luminescence nanoparticles (LPLNP)	ASC	FI
R8 [52]	RRRRRRRRC	Pdots	hMSCs	FI
R8 [58]	–	QD	mASC	FI
TAT [59]	YGRKKRRQRRR	NIR highly fluorescent Pdots	hMSCs	FI
R8 [51]	RRRRRRRRC	NIR highly fluorescent Pdots	hMSCs	FI
TAT [64]	–	TPSi NP	MSC	UI

### Applications of CPPs in fluorescence imaging

Optical imaging has much higher sensitivity, larger throughputs, cheaper and smaller equipment, and multiple detection wavelengths [83]. Currently speaking, fluorescence imaging is a relatively mature and most widely used tracking method.

Fluorescent materials often used in cell and molecular experiments are difficult to apply in animal experiments due to problems such as penetration effect and signal-to-noise ratio. Near-infrared (NIR) luminescent nanoparticles are commonly used imaging luminescent materials in vivo, and luminescent materials combined with CPP have better labeling performance. TAT penetrating peptide bio-conjugated long-lasting luminescence nanoparticles (LPLNP-TAT) was used to track adipose-derived stem cells (ASC) in mice for a long time without continuous external excitation energy [84]. Compared with traditional organic dyes and quantum dots, LPLNP-TAT had near-infrared emission, red light reproducibility, excellent in vivo imaging depth, and higher signal-to-noise ratio, and the complex did not damage the proliferation and differentiation of stem cells. In the study of labeling human mesenchymal stem cells, semiconducting polymer dots (Pdots) coated with CPPs (octa-arginine, R8) had a significant endocytosis and absorption efficiency [85], which was 15 times higher than that of carboxyl Pdots and more than 200 times higher than naked Pdots. After subcutaneous transplantation, Pdot-labeled MSC could be tracked in vitro for 15 generations and within 2 weeks in vivo [52]. Similar reports showed that R8-modified near-infrared fluorescent semiconductor Pdot greatly increased the labeling rate when applied in human mesenchymal stem cells (100 times than that of carboxyl-modified markers), and it could track stem cells within two weeks [51]. Recently, researchers have designed a kind of high fluorescent Pdots with far-red light absorption and NIR emission [80]. After combining with CPP (TAT), the results of flow cytometry showed that the labeled cells improved by about 4 orders of magnitude compared with the control group. In vivo study showed that the stem cells initially accumulated in the lungs and remained there for 7 days.

In addition to using the traditional CPPs, some researchers designed the CPPs according to the specific requirements of the experiment. A study reported an amphiphilic CPP, F6G6(rR)3R2, was designed to transport hydrophobic fluorophores across cell membrane [86]. Three classical thermally activated delayed fluorescence (TADF) molecules, 4CzIPN, NAI-DPAC, and BTZ-DMAC, could self-assemble into well-dispersed nanoparticles (NPs) with F6G6(rR)3R2 in aqueous solution. These NPs showed low cytotoxicity and could penetrate membranes easily. These findings greatly expanded the applications of cell penetrating peptides for delivery of molecules and NPs by only non-covalent interactions, which were more flexible and easier than covalent modifications.

In general, CPP has improved the labeling efficiency of fluorescent particles and has played a role in *in vivo* imaging of small animals to explore the mechanism of stem cells.

### Applications of CPPs in ultrasonic imaging

After X-radiography, UI is now the most common of all the medical imaging technologies, since it is noninvasive, cost-effective, widely available, and allows molecular imaging and real-time guided imaging [87, 88]. One major approach is to label stem cells with UI contrast agents to achieve enhanced imaging of cells [89].

Shengcai et al. [90] designed a cell penetrating peptide (TAT)-conjugated, porous silicon nanoparticle (TPSi NP) loaded with the Wnt3a protein to increase both the cell survival rate and the delivery precision of stem cell transplantation via a combinational theranostic strategy. The volume of PSi NP is relatively large. After TAT is conjugated on its surface, the efficiency of TPSi NP into cells increased from 52.7 to 78.8%. It is confirmed that the intracellular aggregation of TPSi NPs can highly amplify the acoustic scattering of the labeled MSCs, resulting in a 2.3-fold increase in the ultrasound signal compared with that of unlabeled MSCs.

### Applications of CPPs in magnetic resonance imaging

Since being introduced into clinical area, MRI has had a wide range application and become a powerful imaging method. There have been few reports on the experimental use of CPPs in MRI for stem cell tracking. However, among all these reports, CPPs can effectively increase the entering efficiency of magnetic particles and therefore enhance the imaging effect.

As early as 2006, researchers have tried to use CPP (poly-arginine peptides, R9) to modify imaging particles to track rat bone marrow mesenchymal stem cells [91]. In this experiment, fluorescein isothiocyanate (FITC) and Gd were used to label cells, and the results of FI showed that CPP can be internalized into the cytoplasm and nucleus at room temperature, 4 °C and 37 °C; in MRI, Gd was incorporated into the cells by peptide in a time- or concentration-dependent manner, and thereby enhancing the detection effect. In another study [79], three peptides were used to compare the labeling effects on human MSCs (hMSCs). The results showed that compared with the linear MSC-specific peptide EM7 and the cyclic MSC-specific peptide CC9, TAT has both the labeling function and the enhancement of T1 contrast, and is more suitable for stem cell labeling. To explore the biodistribution of cells, CPPs had also been used to help label and track CAR-T cells. In experiments on mice, TAT increased the MRI signal intensity of perfluorocarbon nano-emulsions by 8 times, and the tumor cell killing analysis showed that the markers did not affect cell function and viability [92]. Unfortunately, the application of CPPs to enhance MRI labeling effect of MSCs is still in the early experiments in vitro, although CPPs have been proven to be widely effective in increasing the signal strength of magnetic particles.

In order to further analyze the effects and side effects of CPPs in MRI, we investigated its application in other cells. Intracellular metabolism under the regulation of signal transduction is essence of life activities. In order to achieve transcriptional imaging of mRNA in vivo, Gd was used to label mRNA [59]. The results showed that the use of d-TAT_57-49_ increased the uptake of the contrast agent by the cells. And the contrast agent is mainly located in the vesicles around the nucleus and did not enter the nucleus. In another experiment in 2017 [93], transmission electron microscopy showed that superparamagnetic iron oxide (SPIO)-R11 mainly concentrated on cell vesicles and lysosomes, and did not significantly damage the ultrastructure of cells. In addition, the fluorescent localization of synthetic MRI and fluorescent bimodal probes also showed that the probes were in the vesicles around the nucleus [94]. The above results certificate the safety of CPP in MRI.

The more common application field of MRI is the diagnosis and treatment of tumors and cancers. Cancer has so far been a major problem in the medical world, so research on cancer started earlier and is more in-depth. As a powerful cell penetrating agent, CPPs are widely used in this field. Unlike tracking stem cells injected to the body, tumor tracers need to be more specific to find the aboriginal cell of the body. That is, the contrast agent needs to specifically identify the lesion site. CPP alone cannot meet this requirement, so ACPP or modified CPP was used in magnetic particles. The ACPP and its Gd-loaded dendrimer form (ACPPD-Gd) had been shown to selectively accumulate in tumors (Py8119 cells) and were clearly detected by observers in rodent models (0.86 vs. 0.69, *p* = 0.04) [95]. The cell penetrating phosphor-peptide modified Peptide-NaGdF4 (4.2 nm in diameter) nanodots greatly improved their MRI contrast ability in tumors [96]. In terms of drug delivery, NPs modified with folic acid and ACPP have excellent cancer targeting capabilities and prolonged the half-life of drugs in nude mice [97]. Superparamagnetic iron oxide nanoparticles combined with porphyrin and TAT penetrating peptide promoted the photodynamic effect of mouse enamel melanoma B78-H1 cells [98]. The detection signal and therapeutic effect of the anticancer drug Olsalazine modified by the penetrating peptide RVRR increased by 6.5 times [58]. ACPP-modified anticancer drugs can even improve the permeability of chondrosarcoma [99].

It is worth mentioned that in the experiment to find potential Alzheimer's disease markers, the contrast agent using TAT penetrating peptide can effectively cross the blood–brain barrier of transgenic mice, and showed obvious uptake effect and improved retention time [100]. This result made it possible to track stem cells used in brain diseases treatments.

However, CPPs are not a one-way transmembrane reagent; it may outflow from cells after carrying the contrast agent into the cell in a concentration and time-dependent manner. In response to this feature, the researchers added a cleavage sequence identifiable by the target cell to the labeling particle complex to prevent the escape of the contrast agent. For example, the CPPs on galactose in the probe Gd-DOTA-k(FR)-Gal-CPPs were cleaved in cells expressing β-gal, so that the reporter group was better retained in the cell [94].

## The future of CPPs in stem cell tracking

### Advantages and disadvantages compared with other delivery vehicles

Commonly used molecular delivery systems are divided into biophysics-based technologies and biochemical-based technologies.

Microinjection and electroporation are biophysical methods. Microinjection is mostly used for the delivery of bulky cells, such as animal egg cells [101]. This process bypasses the barriers associated with the delivery of components through the extracellular matrix, cell membrane, and cytoplasm. In addition, microinjection is not limited by the molecular weight of the cargo. However, this method is more intuitive and is limited to specific types of cells; besides, it is difficult to deliver to a large number of cells, especially difficult to apply in vivo. The electroporation method can deliver a large number of target particles into the cell, but the disadvantage is that it damages the cell membrane and affects the cell viability [78]. It is also difficult to apply in in vivo experiments.

Biochemical delivery media include amphiphilic molecules, liposomes, and so on. These two methods are relatively mild and cost less. The disadvantage is that once the liposomal nanoparticle has crossed the cell surface, it is usually encapsulated in endosomes. Cells can very quickly direct the encapsulated contents into the lysosomal pathway, leading to the degradation of all lysosomal contents [102]. This results in a lower conversion rate in the body.

In contrast, the disadvantage of CPP is that it is easily degraded in the body. However, CPP has a higher conversion efficiency than electroporation, has a lower effective penetration concentration and toxicity than liposomes, and can be degraded into harmless amino acids in the body.

### Possible applications of CPP in future clinical trials

As we delve deeper into stem cells, the tremendous role that transplanted stem cells played in the body has been fully recognized. The imaging technology to track transplanted stem cells has matured day by day, and CPPs are undoubtedly a good tool in this technology. It can increase the uptake of tracking particles by stem cells and is non-toxic to cells. After simple modification (addition of a cleavage site that can be recognized by the target cell), the tracking particles can stay in the cell for a long time. Of course, in order to reduce the damage of the tracer particles to the human body in clinical practice, the non-unidirectional transmembrane property of CPPs allows the labeled particles to be better excreted from the body.

At present, many countries have clinical records for the treatment of various diseases with stem cells, and research on the mechanism of stem cells is in full swing. We believe this question will be answered in the near future. What follows may be a larger-scale clinical application of stem cells. In future medical treatment, stem cell therapy may become as common as blood transfusion, but due to uncertain sources and individual differences, we still need to monitor the transplanted cells. At this time, targeting sequences combined with CPPs and labeled particles can be designed according to the characteristics of transplanted cells to achieve effective non-toxic and noninvasive tracking of stem cells in vivo.

## Outlook

Stem cell tracking is a problem worthy of discussion; it determines the basic data in the future clinical. On the one hand, it is necessary to confirm whether stem cells form tumors to evaluate the safety of stem cell therapy; on the other hand, it is necessary to explore the mechanism of stem cells to find effective factors, and lay the foundation for future clinical application. Among various stem cell tracking methods, including all kinds of imaging techniques, CPPs are an effective tool to improve label efficiency.

At present, there are a few of studies on improving the efficiency of stem cell labeling by modifying the imaging particles with CPPs, but CPPs have significant effects in these reports. In fact, since being discovered in the last century, CPPs have been widely used in the delivery of various cargoes, including clinical drugs, therapeutic proteins, and genes [23, 25–29]. Although the mechanism of CPPs penetrating the cell membrane has not been studied clearly, the penetrating effect is certain, and the CPPs used to deliver cargoes have almost no effect on the viability and differentiation potential of stem cells at the working concentration.

There are many types of CPPs, and TAT is the most widely used one because of its versatility. The ways to combine CPPs with imaging nanoparticles include covalent bonding and non-covalent bonding. Covalent bonding requires unique conjugation design for different cargoes, so non-covalent bonding is more widely used. Non-covalent bonding include biotin–streptavidin interaction [103], electrostatic Interactions [57], and metal affinity interactions [103]. Although the non-covalent binding method is more versatile, it is still difficult for biological laboratories that lacking a chemical background to synthesize CPPs-modified imaging nanoparticles. To make matters worse, at present, there are few commercialized imaging nanoparticles modified by CPPs, which may limit the application of CPPs in cell tracking experiments. In view of the universal effectiveness of CPPs, we believe that the commercialization of CPPs-modified imaging nanoparticles is necessary.

## Data Availability

All data generated or analyzed during this study are included in this published article.

## Abbreviations

MRI: Magnetic resonance imaging
FI: Fluorescence imaging
UI: Ultrasound imaging
CPPs: Cell penetrating peptides
ACPPs: Activatable cell penetrating peptides
PET: Positron emission tomography
TAT: Trans-activator of transcription, a kind of cell penetrating peptides
MAP: Model amphipathic peptide, a kind of cell penetrating peptides
R(*n*): Poly(*n*)-arginine, a kind of cell penetrating peptides, “*n*” means number
ASC: Adipose-derived stem cells
MSC: Mesenchymal stem cell
NPs: Nanoparticles
Pdots: Semiconducting polymer dots
NIR: Near-infrared
FITC: Fluorescein isothiocyanate
SPIO: Superparamagnetic iron oxide

## References

[1] Li M, Guo K, Ikehara S (2014). Stem cell treatment for Alzheimer's disease. Int J Mol Sci.

[2] Maclaren RE, Bennett J, Schwartz SD (2016). Gene therapy and stem cell transplantation in retinal disease: the new frontier. Ophthalmology.

[3] Barba M, Di Taranto G, Lattanzi W (2017). Adipose-derived stem cell therapies for bone regeneration. Expert Opin Biol Ther.

[4] Tang Z, Zhang Y, Wang Y (2017). Progress of stem/progenitor cell-based therapy for retinal degeneration. J Transl Med.

[5] Bittle GJ, Morales D, Deatrick KB (2018). Stem cell therapy for hypoplastic left heart syndrome: mechanism, clinical application, and future directions. Circ Res.

[6] Garitaonandia I, Gonzalez R, Sherman G (2018). Novel approach to stem cell therapy in Parkinson's disease. Stem Cells Dev.

[7] Rikhtegar R, Pezeshkian M, Dolati S (2019). Stem cells as therapy for heart disease: iPSCs, ESCs, CSCs, and skeletal myoblasts. Biomed Pharmacother.

[8] Jin J (2017). Stem cell treatments. JAMA.

[9] Tang R, Murray CW, Linde IL (2020). A versatile system to record cell-cell interactions. Elife.

[10] Carneiro ZA, Lima JC, Lopes CD (2019). Heterobimetallic nickel(II) and palladium(II) complexes derived from S-benzyl-N-(ferrocenyl)methylenedithiocarbazate: Trypanocidal activity and interaction with *Trypanosoma cruzi* old yellow enzyme (TcOYE). Eur J Med Chem.

[11] Kaneko T, Sone PP, Zaw SYM (2019). In vivo fate of bone marrow mesenchymal stem cells implanted into rat pulpotomized molars. Stem Cell Res.

[12] Mareninova OA, Jia W, Gretler SR (2020). Transgenic expression of GFP-LC3 perturbs autophagy in exocrine pancreas and acute pancreatitis responses in mice. Autophagy.

[13] Stapornwongkul KS, De Gennes M, Cocconi L (2020). Patterning and growth control in vivo by an engineered GFP gradient. Science.

[14] Markides H, Newell KJ, Rudorf H (2019). Ex vivo MRI cell tracking of autologous mesenchymal stromal cells in an ovine osteochondral defect model. Stem Cell Res Ther.

[15] Tremblay ML, Davis C, Bowen CV (2018). Using MRI cell tracking to monitor immune cell recruitment in response to a peptide-based cancer vaccine. Magn Reson Med.

[16] Martinez-Banderas AI, Aires A, Plaza-Garcia S (2020). Magnetic core-shell nanowires as MRI contrast agents for cell tracking. J Nanobiotechnol.

[17] Nguyen PK, Riegler J, Wu JC (2014). Stem cell imaging: from bench to bedside. Cell Stem Cell.

[18] Vives E, Brodin P, Lebleu B (1997). A truncated HIV-1 Tat protein basic domain rapidly translocates through the plasma membrane and accumulates in the cell nucleus. J Biol Chem.

[19] Kibria G, Hatakeyama H, Ohga N (2011). Dual-ligand modification of PEGylated liposomes shows better cell selectivity and efficient gene delivery. J Control Release.

[20] Morris MC, Depollier J, Mery J (2001). A peptide carrier for the delivery of biologically active proteins into mammalian cells. Nat Biotechnol.

[21] Guidotti G, Brambilla L, Rossi D (2017). Cell-penetrating peptides: from basic research to clinics. Trends Pharmacol Sci.

[22] Derakhshankhah H, Jafari S (2018). Cell penetrating peptides: a concise review with emphasis on biomedical applications. Biomed Pharmacother.

[23] Bohmova E, Machova D, Pechar M (2018). Cell-penetrating peptides: a useful tool for the delivery of various cargoes into cells. Physiol Res.

[24] Gillmeister MP, Betenbaugh MJ, Fishman PS (2011). Cellular trafficking and photochemical internalization of cell penetrating peptide linked cargo proteins: a dual fluorescent labeling study. Bioconjug Chem.

[25] Ramakrishna S, Kwaku Dad AB, Beloor J (2014). Gene disruption by cell-penetrating peptide-mediated delivery of Cas9 protein and guide RNA. Genome Res.

[26] Chen B, Wu C (2018). Cationic cell penetrating peptide modified SNARE protein VAMP8 as free chains for gene delivery. Biomater Sci.

[27] Oba M, Kato T, Furukawa K (2016). A Cell-penetrating peptide with a guanidinylethyl amine structure directed to gene delivery. Sci Rep.

[28] Lin W, Xie X, Yang Y (2016). Thermosensitive magnetic liposomes with doxorubicin cell-penetrating peptides conjugate for enhanced and targeted cancer therapy. Drug Deliv.

[29] Bolhassani A, Jafarzade BS, Mardani G (2017). In vitro and in vivo delivery of therapeutic proteins using cell penetrating peptides. Peptides.

[30] Jones SW, Christison R, Bundell K (2005). Characterisation of cell-penetrating peptide-mediated peptide delivery. Br J Pharmacol.

[31] Boussoufi F, Gallon SMN, Chang R (2018). Synthesis and study of cell-penetrating peptide-modified gold nanoparticles. Int J Nanomedicine.

[32] Elliott G, Ohare P (1997). Intercellular trafficking and protein delivery by a herpesvirus structural protein. Cell.

[33] De Coupade C, Fittipaldi A, Chagnas V (2005). Novel human-derived cell-penetrating peptides for specific subcellular delivery of therapeutic biomolecules. Biochem J.

[34] Jain A, Yadav BK, Chugh A (2015). Marine antimicrobial peptide tachyplesin as an efficient nanocarrier for macromolecule delivery in plant and mammalian cells. FEBS J.

[35] Pooga M, Soomets U, Hallbrink M (1998). Cell penetrating PNA constructs regulate galanin receptor levels and modify pain transmission in vivo. Nat Biotechnol.

[36] Tunnemann G, Ter-Avetisyan G, Martin RM (2008). Live-cell analysis of cell penetration ability and toxicity of oligo-arginines. J Pept Sci.

[37] Derossi D, Joliot AH, Chassaing G (1994). The third helix of the Antennapedia homeodomain translocates through biological membranes. J Biol Chem.

[38] Brock R (2014). The uptake of arginine-rich cell-penetrating peptides: putting the puzzle together. Bioconjug Chem.

[39] Tunnemann G, Martin RM, Haupt S (2006). Cargo-dependent mode of uptake and bioavailability of TAT-containing proteins and peptides in living cells. FASEB J.

[40] Rothbard JB, Jessop TC, Wender PA (2005). Adaptive translocation: the role of hydrogen bonding and membrane potential in the uptake of guanidinium-rich transporters into cells. Adv Drug Deliv Rev.

[41] Herce HD, Garcia AE (2007). Molecular dynamics simulations suggest a mechanism for translocation of the HIV-1 TAT peptide across lipid membranes. Proc Natl Acad Sci USA.

[42] Ferrari A, Pellegrini V, Arcangeli C (2003). Caveolae-mediated internalization of extracellular HIV-1 tat fusion proteins visualized in real time. Mol Ther.

[43] Kaplan IM, Wadia JS, Dowdy SF (2005). Cationic TAT peptide transduction domain enters cells by macropinocytosis. J Control Release.

[44] Richard JP, Melikov K, Brooks H (2005). Cellular uptake of unconjugated TAT peptide involves clathrin-dependent endocytosis and heparan sulfate receptors. J Biol Chem.

[45] Soler M, Gonzalez-Bartulos M, Soriano-Castell D (2014). Identification of BP16 as a non-toxic cell-penetrating peptide with highly efficient drug delivery properties. Org Biomol Chem.

[46] Randhawa HK, Gautam A, Sharma M (2016). Cell-penetrating peptide and antibiotic combination therapy: a potential alternative to combat drug resistance in methicillin-resistant *Staphylococcus aureus*. Appl Microbiol Biotechnol.

[47] Budagavi DP, Chugh A (2018). Antibacterial properties of Latarcin 1 derived cell-penetrating peptides. Eur J Pharm Sci.

[48] Ramsey JD, Flynn NH (2015). Cell-penetrating peptides transport therapeutics into cells. Pharmacol Ther.

[49] Zou L, Peng Q, Wang P (2017). Progress in research and application of HIV-1 TAT-derived cell-penetrating peptide. J Membr Biol.

[50] Chen LJ, Zhao X, Yan XP (2019). Cell-penetrating peptide-functionalized persistent luminescence nanoparticles for tracking J774A1 macrophages homing to inflamed tissues. ACS Appl Mater Interfaces.

[51] Chen D, Li Q, Meng Z (2017). Bright polymer dots tracking stem cell engraftment and migration to injured mouse liver. Theranostics.

[52] Meng Z, Guo L, Li Q (2017). Peptide-coated semiconductor polymer dots for stem cells labeling and tracking. Chemistry.

[53] Suchy M, Ta R, Li AX (2010). A paramagnetic chemical exchange-based MRI probe metabolized by cathepsin D: design, synthesis and cellular uptake studies. Org Biomol Chem.

[54] Drexelius M, Reinhardt A, Grabeck J (2021). Multistep optimization of a cell-penetrating peptide towards its antimicrobial activity. Biochem J.

[55] Mishra R, Su W, Pohmann R (2009). Cell-penetrating peptides and peptide nucleic acid-coupled MRI contrast agents: evaluation of cellular delivery and target binding. Bioconjug Chem.

[56] Endres PJ, Macrenaris KW, Vogt S (2008). Cell-permeable MR contrast agents with increased intracellular retention. Bioconjug Chem.

[57] Upadhya A, Sangave PC (2016). Hydrophobic and electrostatic interactions between cell penetrating peptides and plasmid DNA are important for stable non-covalent complexation and intracellular delivery. J Pept Sci.

[58] Yuan Y, Zhang J, Qi X (2019). Furin-mediated intracellular self-assembly of olsalazine nanoparticles for enhanced magnetic resonance imaging and tumour therapy. Nat Mater.

[59] Su W, Mishra R, Pfeuffer J (2007). Synthesis and cellular uptake of a MR contrast agent coupled to an antisense peptide nucleic acid–cell- penetrating peptide conjugate. Contrast Media Mol Imaging.

[60] Cousins MJ, Pickthorn K, Huang S (2013). The safety and efficacy of KAI-1678- an inhibitor of epsilon protein kinase C (epsilonPKC)-versus lidocaine and placebo for the treatment of postherpetic neuralgia: a crossover study design. Pain Med.

[61] Eshraghi AA, Aranke M, Salvi R (2018). Preclinical and clinical otoprotective applications of cell-penetrating peptide D-JNKI-1 (AM-111). Hear Res.

[62] Staecker H, Jokovic G, Karpishchenko S (2019). Efficacy and safety of AM-111 in the treatment of acute unilateral sudden deafness-a double-blind, randomized, placebo-controlled phase 3 study. Otol Neurotol.

[63] Jankovic J, Truong D, Patel AT (2018). Injectable daxibotulinumtoxin A in cervical dystonia: a phase 2 dose-escalation multicenter study. Mov Disord Clin Pract.

[64] Chiquet C, Aptel F, Creuzot-Garcher C (2017). Postoperative ocular inflammation: a single subconjunctival injection of XG-102 compared to dexamethasone drops in a randomized trial. Am J Ophthalmol.

[65] Warso MA, Richards JM, Mehta D (2013). A first-in-class, first-in-human, phase I trial of p28, a non-HDM2-mediated peptide inhibitor of p53 ubiquitination in patients with advanced solid tumours. Br J Cancer.

[66] Lulla RR, Goldman S, Yamada T (2016). Phase I trial of p28 (NSC745104), a non-HDM2-mediated peptide inhibitor of p53 ubiquitination in pediatric patients with recurrent or progressive central nervous system tumors: a pediatric brain tumor consortium study. Neuro Oncol.

[67] Savariar EN, Felsen CN, Nashi N (2013). Real-time in vivo molecular detection of primary tumors and metastases with ratiometric activatable cell-penetrating peptides. Cancer Res.

[68] Miampamba M, Liu J, Harootunian A (2017). Sensitive in vivo visualization of breast cancer using ratiometric protease-activatable fluorescent imaging agent, AVB-620. Theranostics.

[69] Unkart JT, Chen SL, Wapnir IL (2017). Intraoperative tumor detection using a ratiometric activatable fluorescent peptide: a first-in-human phase 1 study. Ann Surg Oncol.

[70] Xu J, Khan AR, Fu M (2019). Cell-penetrating peptide: a means of breaking through the physiological barriers of different tissues and organs. J Control Release.

[71] Deng X, Mai R, Zhang C (2021). Discovery of novel cell-penetrating and tumor-targeting peptide-drug conjugate (PDC) for programmable delivery of paclitaxel and cancer treatment. Eur J Med Chem.

[72] Ha M, Nam SH, Sim K (2021). Highly efficient photothermal therapy with cell-penetrating peptide-modified bumpy Au triangular nanoprisms using low laser power and low probe dose. Nano Lett.

[73] Nam SH, Jang J, Cheon DH (2021). pH-activatable cell penetrating peptide dimers for potent delivery of anticancer drug to triple-negative breast cancer. J Control Release.

[74] Zhang YF, Wu YF, Lan TJ (2020). Codelivery of anticancer drug and photosensitizer by PEGylated graphene oxide and cell penetrating peptide enhanced tumor-suppressing effect on osteosarcoma. Front Mol Biosci.

[75] Sagnella SM, Mccarroll JA, Kavallaris M (2014). Drug delivery: beyond active tumour targeting. Nanomedicine.

[76] Kawasaki Y, Akiyama T (2015). Tumor microenvironment: promising therapeutic target. Nihon Rinsho.

[77] Yoo J, Sanoj Rejinold N, Lee D (2017). Protease-activatable cell-penetrating peptide possessing ROS-triggered phase transition for enhanced cancer therapy. J Control Release.

[78] Zhang D, Wang J, Xu D (2016). Cell-penetrating peptides as noninvasive transmembrane vectors for the development of novel multifunctional drug-delivery systems. J Control Release.

[79] Cao L, Li B, Yi P (2014). The interplay of T1- and T2-relaxation on T1-weighted MRI of hMSCs induced by Gd-DOTA-peptides. Biomaterials.

[80] Yuan Y, Zhang Z, Hou W (2020). In vivo dynamic cell tracking with long-wavelength excitable and near-infrared fluorescent polymer dots. Biomaterials.

[81] Wolfe JM, Fadzen CM, Holden RL (2018). Perfluoroaryl bicyclic cell-penetrating peptides for delivery of antisense oligonucleotides. Angew Chem Int Ed Engl.

[82] Yu Y, Zu C, He D (2021). pH-dependent reversibly activatable cell-penetrating peptides improve the antitumor effect of artemisinin-loaded liposomes. J Colloid Interface Sci.

[83] Wu TJ, Tzeng YK, Chang WW (2013). Tracking the engraftment and regenerative capabilities of transplanted lung stem cells using fluorescent nanodiamonds. Nat Nanotechnol.

[84] Wu SQ, Chi CW, Yang CX (2016). Penetrating peptide-bioconjugated persistent nanophosphors for long-term tracking of adipose-derived stem cells with superior signal-to-noise ratio. Anal Chem.

[85] Yukawa H, Kagami Y, Watanabe M (2010). Quantum dots labeling using octa-arginine peptides for imaging of adipose tissue-derived stem cells. Biomaterials.

[86] Zhu Z, Tian D, Gao P (2018). Cell-penetrating peptides transport noncovalently linked thermally activated delayed fluorescence nanoparticles for time-resolved luminescence imaging. J Am Chem Soc.

[87] Wells PN, Liang HD (2011). Medical ultrasound: imaging of soft tissue strain and elasticity. J R Soc Interface.

[88] Schutt EG, Klein DH, Mattrey RM (2003). Injectable microbubbles as contrast agents for diagnostic ultrasound imaging: the key role of perfluorochemicals. Angew Chem Int Ed Engl.

[89] Lyu Y, Zhen X, Miao Y (2017). Reaction-based semiconducting polymer nanoprobes for photoacoustic imaging of protein sulfenic acids. ACS Nano.

[90] Qi S, Zhang P, Ma M (2019). Cellular internalization-induced aggregation of porous silicon nanoparticles for ultrasound imaging and protein-mediated protection of stem cells. Small.

[91] Liu M, Guo YM, Yang JL (2006). Application of cell penetrating peptide in magnetic resonance imaging of bone marrow mesenchymal stem cells. Acta Biochim Biophys Sin (Shanghai).

[92] Hingorani DV, Chapelin F, Stares E (2020). Cell penetrating peptide functionalized perfluorocarbon nanoemulsions for targeted cell labeling and enhanced fluorine-19 MRI detection. Magn Reson Med.

[93] Ding C, Wu K, Wang W (2017). Synthesis of a cell penetrating peptide modified superparamagnetic iron oxide and MRI detection of bladder cancer. Oncotarget.

[94] Keliris A, Ziegler T, Mishra R (2011). Synthesis and characterization of a cell-permeable bimodal contrast agent targeting beta-galactosidase. Bioorg Med Chem.

[95] Malone CD, Olson ES, Mattrey RF (2015). Tumor detection at 3 tesla with an activatable cell penetrating peptide dendrimer (ACPPD-Gd), a T1 magnetic resonance (MR) molecular imaging agent. PLoS ONE.

[96] Chen H, Li X, Liu F (2017). Renal clearable peptide functionalized NaGdF4 Nanodots for high-efficiency tracking orthotopic colorectal tumor in mouse. Mol Pharm.

[97] Gao P, Mei C, He L (2018). Designing multifunctional cancer-targeted nanosystem for magnetic resonance molecular imaging-guided theranostics of lung cancer. Drug Deliv.

[98] Thandu M, Rapozzi V, Xodo L (2014). "Clicking" porphyrins to magnetic nanoparticles for photodynamic therapy. ChemPlusChem.

[99] Vares G, Jallet V, Matsumoto Y (2020). Functionalized mesoporous silica nanoparticles for innovative boron-neutron capture therapy of resistant cancers. Nanomedicine.

[100] Ta R, Suchy M, Tam JH (2013). A dual magnetic resonance imaging/fluorescent contrast agent for Cathepsin-D detection. Contrast Media Mol Imaging.

[101] Reissmann S (2014). Cell penetration: scope and limitations by the application of cell-penetrating peptides. J Pept Sci.

[102] Lino CA, Harper JC, Carney JP (2018). Delivering CRISPR: a review of the challenges and approaches. Drug Deliv.

[103] Nitin N, Laconte L, Rhee WJ (2009). Tat peptide is capable of importing large nanoparticles across nuclear membrane in digitonin permeabilized cells. Ann Biomed Eng.

